# Texas Sanitary Association

**Published:** 1890-03

**Authors:** 


### TEXAS SANITARY ASSOCIATION.

The Texas Sanitary Convention met in Dallas, on March 13. The President of the Association being absent, Dr. T. C. Osborn, of Cleburne, was elected to the chair. Rev. Dr. Buckner, of Dallas, offered prayer.

At the evening session officers were elected as follows: Drs. T. C. Osborn, of Cleburne, President; C. M. Rosser, First Vice President; J. C. Loggins, of Ennis, Second Vice President; D. A. Poynor, Third Vice President; G. W. Stewart, Secretary, and A. M. Elmore, Treasurer.

Several interesting and exhaustive papers were read and referred to the Committee on Publication.

The following was adopted:

"Resolved, That the President and Vice President of the Association be requested to organize local associations, one or more for each county in the State, to hold quarterly meetings and make such reports to the corresponding secretary as the Association may deem necessary in the interest of sanitation."

The Association then adjourned to meet in Dallas on the second Monday in March of next year.

---

DILLON'S MILLS, VA., March 27, 1887.

I have been using Lactopeptine in my practice for a number of years, but have never used any remedy for the nausea and vomiting in children which has given me as much satisfaction or been attended with such uniformly good results as your Lactopeptine Elixir. It is put up in beautiful style, and easy of administration to the most delicate child.

I used the Lactopeptine Syrup with Phosphates in the case of an aged patient in the last stages of phthisis, whose stomach rejected almost everything he ate, and after taking a few doses, his stomach became quiet, and he continued the use of it until he had taken all, after which the vomiting did not return. I thank you heartily for the samples, and shall continue to use your preparations. I remain, yours truly,

M. T. GREER, M. D.

